# Why Economic Analysis of Health System Improvement Interventions Matters

**DOI:** 10.3389/fpubh.2016.00218

**Published:** 2016-10-11

**Authors:** Edward Ivor Broughton, Lani Marquez

**Affiliations:** ^1^Johns Hopkins Bloomberg School of Public Health, Baltimore, MD, USA; ^2^USAID Applying Science to Strengthen and Improve Systems (ASSIST) Project, University Research Co., LLC, Bethesda, MD, USA

**Keywords:** health system improvement, economic analysis, cost-effectiveness, medical errors

## Abstract

There is little evidence to direct health systems toward providing efficient interventions to address medical errors, defined as an unintended act of omission or commission or one not executed as intended that may or may not cause harm to the patient but does not achieve its intended outcome. We believe that lack of guidance on what is the most efficient way to reduce medical errors and improve the quality of health-care limits the scale-up of health system improvement interventions. Challenges to economic evaluation of these interventions include defining and implementing improvement interventions in different settings with high fidelity, capturing all of the positive and negative effects of the intervention, using process measures of effectiveness rather than health outcomes, and determining the full cost of the intervention and all economic consequences of its effects. However, health system improvement interventions should be treated similarly to individual medical interventions and undergo rigorous economic evaluation to provide actionable evidence to guide policy-makers in decisions of resource allocation for improvement activities among other competing demands for health-care resources.

## Introduction

It is clear that medical errors carry a very high burden of disease (1) and are considered a problem to be addressed by the health system as much as physiological conditions, such as ischemic heart disease (IHD) and stroke, the two leading causes of mortality worldwide (2). Interventions, such as antihypertensive and antiplatelet medications, are generally established as sufficiently cost-effective to reduce the risk of IHD and stroke. However, there is little evidence to direct health systems toward providing the most efficient interventions to address medical errors (3, 4). Medical errors are defined as an unintended act of omission or commission or an act not executed as intended that may or may not cause harm to the patient but does not achieve its intended outcome (5, 6). This definition encompasses care that fails to meet evidence-based standards by omitting steps or elements of care that have been deemed to be part of accepted international practice, even if it does not cause direct harm to the patient. Seeing substandard care in this light – that is, as a medical error – is important to draw attention to the costs to the health system and to patients and communities of poor quality care. Economic analyses, by which we mean cost-effectiveness analysis, cost–benefit analysis, and cost–utility analysis, are prevalent in the biomedical literature for individual medical interventions (7). We propose in this paper that the same economic analysis used in individual medical interventions also be routinely used to determine which interventions at the health system level are the most efficient in addressing medical errors.

## Evaluation of Improvement Interventions to Decrease Errors

There are many reports of evaluations of interventions to improve health service delivery to decrease the incidence of errors that lead to adverse health events (7–9) and to increase adherence to evidence-based standards (10–12), including in low- and middle-income countries (13–15). While the number of these is not as high as for interventions dealing directly with individual patient conditions, there is now a significant body of evidence of the effectiveness of such interventions. However, there are far fewer economic analyses of these health system improvement interventions (HSIIs) that decrease errors and improve quality (16). As a result, there is little evidence to guide health delivery managers and policy makers on what is the most efficient way to reduce adverse events and improve quality (3, 16, 17).

Because HSIIs are often complex social interventions, seeking to influence both provider and patient behaviors as well as health-care organization, economic evaluations of these interventions – including cost-effectiveness, cost–utility, and cost–benefit analyses – can be more difficult to conduct and more complicated to interpret their results. In this paper, we argue that complexity is no excuse not to do such evaluations. Evidence-based HSIIs are urgently needed to best serve those who entrust their welfare to health service providers and the system in which they work. Cost-effectiveness analyses, cost-utility analyses, and other economic evaluations inform decision-makers on how efficient and therefore how worthwhile such interventions are compared to other uses of scarce resources.

We outline challenges to conducting economic analysis on HSIIs to reduce health care-associated errors and increase adherence to evidence-based standards and discuss ways these can be overcome or accommodated (Table 1). For illustration, we discuss a cost-effectiveness analysis of the use of uterotonics during the third stage of labor to reduce the risk of post-partum hemorrhage (PPH) in Senegal as an example of a simple, well-defined individual medical intervention that has a single basic intended effect (18) in contrast to the cost-effectiveness analysis of a HSII to improve compliance with essential obstetric and newborn care (including the use of uterotonics in the third stage of labor) in Niger (19). The latter considers the system of delivery of care rather than just the administration of a specific treatment to a patient group. For simplicity, this paper considers empirical evaluations of HSIIs, but the principles apply as well to modeling studies based on hypothetical interventions.

**Table 1:** **Measurement challenges and potential solutions in conducting economic evaluations of health system improvement interventions**.

Measurement challenge	Potential solutions
Defining intervention	Empirically define intervention; use modeling, Monte Carlo Simulation, and sensitivity analysis to account for low fidelity to intervention
Defining effectiveness	Measure all positive and potentially negative effects (balancing measures) as feasible; report all effects clearly
Process measures versus outcomes	Use epidemiological modeling (e.g., LiST Tool) to convert processes to outcomes; use DALYs or QALYs for all outcomes
Costing the intervention	Collect cost data prospectively; secure agreement with improvement implementers to report all resources used
Economic consequences of effects	Collect primary data if possible; report assumptions made and account for them in modeling

## Defining the Intervention

Health system improvement interventions to reduce errors and improve health service quality often have a degree of complexity and adaptation to local context that individual medical interventions usually do not. There may be multiple components in HSIIs, such as training of health-care providers, providing job aids and reminders to reinforce good practice, restoring essential medication supply chains, improving patient record-keeping, supervision or coaching, and enhancing facility infrastructure (20), as was the case in the Niger example. As HSIIs are often implemented at the service delivery level, the actual activities that comprise the intervention may vary substantially depending on the context. This is in contrast with individual medical interventions in which there is generally high fidelity to a single described intervention. In the Senegal case, the intervention consisted only of a standardized dose of either misoprostol or oxytocin, and the outcome of interest was a drop in hemoglobin level of the mother or a referral to a hospital for PPH.

A solution suggested for this problem is to define explicitly all the components of the HSII being implemented and the context in which they were introduced. Then, if it is useful to consider the same intervention in different settings or under different circumstances in the same settings, adjustments can be made in the expected results through the use of different scenarios in decision trees and well-established statistical techniques, such as Monte Carlo simulations and sensitivity analyses. Such modeling could reduce precision in the results but would provide a more objective basis for estimating likely benefits under different scenarios.

## Defining the Effects

Assuming success, HSIIs often have more than one positive effect on health outcomes of those receiving care from the heath provider unit involved. In the Niger example, the primary effect was reduced maternal morbidity and mortality. However, improvements in obstetric care were also accompanied by improvements in newborn care and therefore, a likely reduction in newborn mortality and morbidity, though this was not captured in the study.

A solution for this problem is to consider as many of the positive effects as can be feasibly measured in the analysis, perform separate analyses on each of them, and then report all outcomes as a composite score. In the example, if the intervention decreased the risk of newborn asphyxia and sepsis as well as maternal PPH, then the cost-effectiveness result could be reported as “for each $100 spent, there was a decrease of X_1_ cases of PPH and X_2_ cases of neonatal asphyxia and X_3_ cases of newborn sepsis. Alternatively, it could be reported that for a total of $Y_2_ in a facility that served Z_1_ people, there were X_4_ fewer cases of PPH, X_5_ fewer cases of neonatal asphyxia, and X_6_ fewer cases of newborn sepsis.”

Conversely, with implementation of a HSII there may be unintended negative consequences of the intervention. In our example, increased health worker attention toward improving delivery of essential maternal and newborn care may lead health workers to neglect other parts of their duties and consequently a deterioration in care quality for patients not receiving service for the focus condition.

The way to deal with this is to measure all the unintended negative consequences that are feasible to identify and include them in the analysis. This is standard practice in individual medical intervention economic evaluations – to measure the severity and frequency of adverse events and to include these in the analyses.

Also, when considering multiple different health outcomes, both positive and negative, from an HSII, it is useful to denominate the outcomes in burden of disease measures, such as disability-adjusted life years (DALYs) or quality-adjusted life years (QALYs). Converting different outcomes to a single unit also has an advantage of permitting comparisons among HSIIs to identify where the health system as a whole will reap the most benefit from investment in HSIIs.

## Process Measures Instead of Outcome Measures

Often in HSIIs, it is more feasible and accurate to measure the effect of the intervention in process measures rather than patient health outcomes. In the Niger example, measurement of compliance to evidence-based standards of care for active management of the third stage of labor and essential newborn care was easier than measuring some maternal and neonatal outcomes, especially when considering relatively rare outcomes such as maternal mortality from PPH. This can also be the case with individual medical interventions. One possible solution is to measure the process indicators as is feasible but then use epidemiological modeling to relate those process measures to tangible health outcomes using the best available evidence of the link between the process and ultimate outcomes. Models, such as the Lives Saved Tool (LiST) (21), offer estimates for many priority facility- and community-based health interventions. Any uncertainty around the estimate of the link between processes and outcomes can be reflected in the model again using sensitivity analysis and Monte Carlo simulation as indicated.

## Costing of the Intervention

Given the lack of fidelity to the prescribed intervention that can occur with HSIIs, determining the intervention’s cost can be difficult. The most accurate method is to collect data on costs prospectively, being sure to record all of the resources consumed, including changes to health providers’ time, compared to the scenario in which the HSII is not implemented. However, retrospective reviews of accounting records can produce reasonable cost estimates. Assumptions often need to be made in calculating accurately the opportunity cost of applying HSIIs, and it is important that these assumptions be stated explicitly in the report of the evaluation. Organizations implementing HSIIs may also be protective of cost data to protect their commercial interests. Therefore, it can be useful for those commissioning the intervention to make such reporting a requirement of conducting the work. The ongoing costs of maintaining the improved level of service often need to be estimated with imperfect information, and any assumptions made here should also be stated transparently in the evaluation.

## Costing of Effects

Given that HSIIs for medical error reduction often involve multiple different health outcomes, determining the economic impact can add significantly to the complexity of data collection and modeling. In the Niger example, the cost from the health system perspective of adverse events such as mild, moderate, or severe PPH, mismanaged obstructed labor, retained placenta and other adverse outcomes were calculated based on daily hospital costs and average lengths of stay. Calculating the economic impact from the societal perspective would have involved including the cost to mothers and their families of additional time in hospital and other related expenses. In such low-income settings, secondary economic data may be scarce, and primary data expensive and difficult to collect. Assumptions on economic impact may be needed, and this is acceptable if reported transparently in the evaluation write-up.

## Discussion and Conclusion

Individual medical interventions often involve a single medication (or class of medications) or medical device. There is often a commercial interest in providing evidence of their effectiveness or cost-effectiveness in treating a specific medical condition. Many health systems, such as the UK’s National Health Service and Australia’s Medicare, mandate that individual medical interventions must undergo rigorous cost-effectiveness analyses before there is financial coverage for them to be used to treat the specific medical conditions. This is not the case for HSIIs, where the intervention is generally not considered a marketable product and there are no mandates to prove their effectiveness or efficiency before they are employed. Consequently, the resources available to thoroughly evaluate their value in improving health are much smaller. Yet, HSIIs do consume resources – resources that could be invested in other interventions to benefit patients or providers. We believe that the donors and national governments should insist on greater transparency in the resources devoted to reducing medical errors and substandard care.

The solution to this is for health system policy-makers to mandate that economic analyses of HSIIs be conducted to produce evidence that an HSII is acceptably cost-effective or cost-saving before resources are expended to implement it, particularly at large scale. It is clear that there are multiple competing demands on health-care resources in all settings, and by definition this is especially the case in low- and middle-income settings. It could be viewed as ethically dubious in such settings to implement an HSII without examination of its economic consequences because, without knowing if the intervention is affordable, sustainable, or acceptably efficient, information on its effectiveness alone is not useful. As with individual medical interventions, studies of HSII effectiveness are necessary but not sufficient to recommend implementing the intervention in a given setting.

This is not to ignore the additional difficulties of performing economic analyses on complex social system interventions. It adds to the cost and time required to evaluate the program and the expertise required to carry it out. However, these factors cannot be excuses to omit such an important aspect of determining the applicability of the HSII. There is no place where health-care resources are inexhaustible and where difficult resource allocation decisions do not need to be made. Economic analysis of HSIIs is a vital part of the science of improvement that helps health systems advance.

## Author Contributions

EB conceived of the idea and drafted the initial version of the manuscript. LM contributed substantively to the writing and editing the manuscript and responding to reviewer comments.

## Conflict of Interest Statement

The authors declare that the research was conducted in the absence of any commercial or financial relationships that could be construed as a potential conflict of interest.
